# Plasma-negative, Renal-limited Cryofibrinogen-associated Glomerulonephritis: A Unique Case Report

**DOI:** 10.1016/j.xkme.2025.101017

**Published:** 2025-04-25

**Authors:** Jarrad A. Hopkins, Ann Nguyen-Hoang, John Brealey, Pravin Hissaria, Jola Kapojos

**Affiliations:** 1 Faculty of Health and Medical Sciences, The University of Adelaide, Adelaide, South Australia; 2 Renal Unit, Division of Medicine, Lyell McEwin Hospital, South Australia, Australia; 3 Department of Anatomical Pathology, SA Pathology, Adelaide, Australia; 4 Department of Immunology, SA Pathology, Adelaide, Australia

**Keywords:** Biopsy-proven, cryofibrinogen, glomerulonephritis

## Abstract

Cryofibrinogen-associated glomerulonephritis is characterized by membranoproliferative glomerulonephritis without immunoglobulin deposition and unique ultrastructural features. This case report presents a 63-year-old man with renal-limited cryofibrinogen-associated glomerulonephritis, with negative plasma cryofibrinogen levels. His medical history included metallic aortic valve replacement and long-term anticoagulation therapy. Clinical examination revealed no cutaneous manifestations or thrombotic events. Initial laboratory investigations showed severe kidney dysfunction, but negative results for plasma cryofibrinogen, serum cryoglobulin, and a comprehensive autoimmune, infective, and malignancy panel. Kidney biopsy revealed mesangiocapillary glomerulonephritis with focal vasculitis and significant interstitial fibrosis, and electron microscopy identified double-walled microtubules consistent with cryofibrinogen. Our patient was managed without immunosuppressive therapy due to significant kidney scarring and absence of extra-renal manifestations. To our knowledge, this case describes the first report of cryofibrinogen-associated glomerulonephritis in the absence of detectable cryofibrinogen in serum, with diagnosis relying on ultrastructural findings. Differential diagnoses such as immunotactoid glomerulonephritis were considered but ruled out based on morphological characteristics. This case adds to the limited literature on renal-limited cryofibrinogen and emphasizes the necessity for thorough investigation including electron microscopy assessment of kidney biopsies to ascertain the diagnosis.

Cryofibrinogen-associated glomerulonephritis is described as membranoproliferative glomerulonephritis in the absence of immunoglobulin deposition, with weak glomerular segmental capillary wall fibrinogen deposition and ultrastructural organized deposits of multilayered large-bore tubular and fine fibrillary structures in the matrix.^1^

Cryofibrinogenemia may be essential (primary) or secondary to infections, solid-organ and hematologic malignancies, autoimmune diseases, or other processes.^1^ Cutaneous or thrombotic symptomatology of cryofibrinogen-associated conditions is present in most (80%) cases, and may include purpura, ulcerations, livedo reticularis, or Raynaud’s.^2^^,^^3^ Kidney involvement in cryofibrinogen-related conditions has been reported in 11%-13% of cases.^4^^,^^5^ The known literature in these cases describes a positive circulating cryofibrinogen when the blood is correctly tested. The cryofibrinogen precipitates form after plasma cooling, but this does not happen with serum.^6^ We describe a unique case of plasma-negative, renal-limited cryofibrinogen glomerulonephritis.

## Case Report

A 63-year-old White man presented to his general practitioner with fatigue and dyspnea. His past medical history included metallic aortic value replacement 6 years ago for aortic stenosis, hypertension, and hyperlipidemia. Relevant medications included warfarin (Marevan), amlodipine, valsartan, hydrochlorothiazide, and atorvastatin. He was found to have a systolic blood pressure of 180 mm Hg and no rash, ulcerations, lymphadenopathy, or edema. There were no clinical manifestations of cryofibrinogen including no cutaneous signs or thromboembolic phenomenon, acknowledging he was on long-standing therapeutic anticoagulation. He had severe kidney impairment with serum creatinine 347 μmol/L (last result 100 μmol/L 4 years prior), urea 15.5 mmol/L, potassium 3.6 mmol/L, albumin 35 g/L, C-reactive protein 1.8 mg/L, and hemoglobin 92 g/L. Urine assessment showed urine protein-creatinine ratio 208 mg/mmol and urinary red blood cells 583. Further 24-hour urinary protein was subnephrotic 1.6 g in 24 hours.

He had no detectable antibodies to myeloperoxidase, proteinase 3, glomerular basement membrane, hepatitis C virus, hepatitis B virus surface antigen, and HIV. Furthermore, antinuclear antibody, double-stranded DNA antibody, extractable nuclear antigen antibody, and rheumatoid factor were negative. Serum C3 and C4 were normal. Serum protein electrophoresis showed 2 g/L IgG kappa paraprotein with serum κ and λ free light chains of 40.14 mg/L (reference, 3.3-19.4 mg/L) and 24.67 mg/L (reference 5.7-26.3 mg/L), respectively, with κ/λ free light chain ratio of 1.63 (reference, 0.26-1.65 κ/λ), consistent with kidney impairment. Serum cryoglobulin and plasma cryofibrinogen were negative at presentation, 1 week, and 2 months later.

Computed tomography myeloma skeletal survey was negative for bony lytic lesions. Investigations for infective endocarditis were negative, including transesophageal echocardiogram, blood cultures, and specific testing for *Coxiella burnetti* and *Bartonella henselae*.

**Figure 1:**
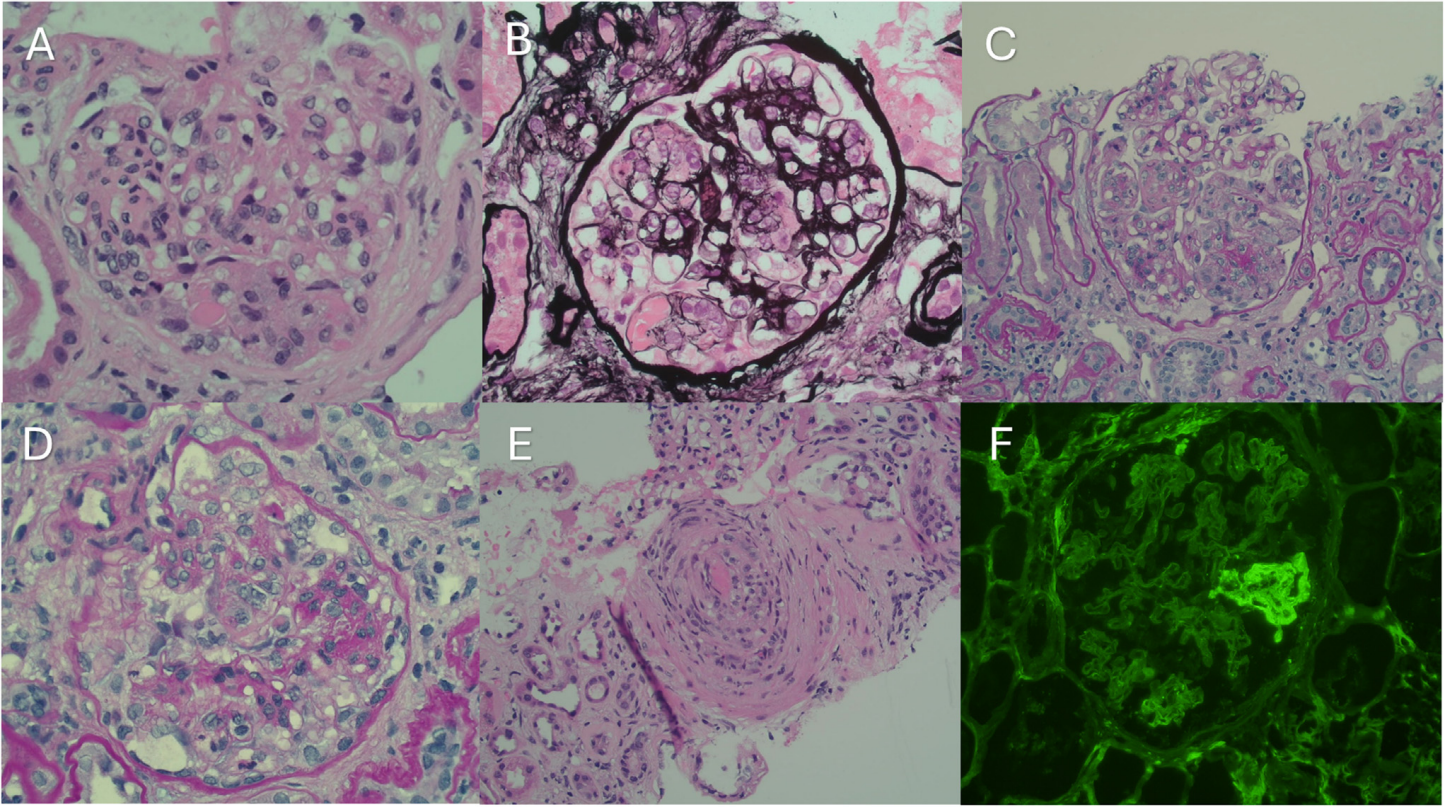
A rounded eosinophilic deposit, resembling a “pseudothrombus,” was seen within a glomerular capillary loop on hemotoxylin and eosin (H&E) (A) and periodic acid–Schiff/methanamine silver staining (B). Endocapillary lesions formed by inflammatory cells and swollen endothelial cells plugging capillary loops were readily seen, as was focal mesangial hypercellularity (C, alcian blue/periodic acid–Schiff; D, H&E), with rare double contours (not pictured), indicating a membranoproliferative pattern of injury. A focus of acute vasculitis with intraluminal thrombus was also noted on H&E staining (E). Immunofluorescent labeling for fibrinogen, showing segmental glomerular capillary loop labeling (F). Immunoglobulins were effectively nonspecific weak to negative, while C3 showed weak granular labeling (not pictured).

Kidney biopsy (Fig 1) showed mesangiocapillary glomerulonephritis and a single rounded glomerular intracapillary eosinophilic deposit, resembling a “pseudothombus.” A focus of active vasculitis was noted. There was severe background interstitial fibrosis and tubular atrophy, without features to suggest separate primary tubulointerstitial pathology.

By immunofluorescence, the immunoglobulins were either negative or showed only weak nonspecific staining. C3 showed weak granular mesangial staining. Fibrinogen exhibited weak but convincing segmental labeling (Fig 1F). Kappa and lambda were weak with no restricted expression. These results were corroborated with immunofluorescence performed after antigen retrieval/protease digestion.

**Figure 2:**
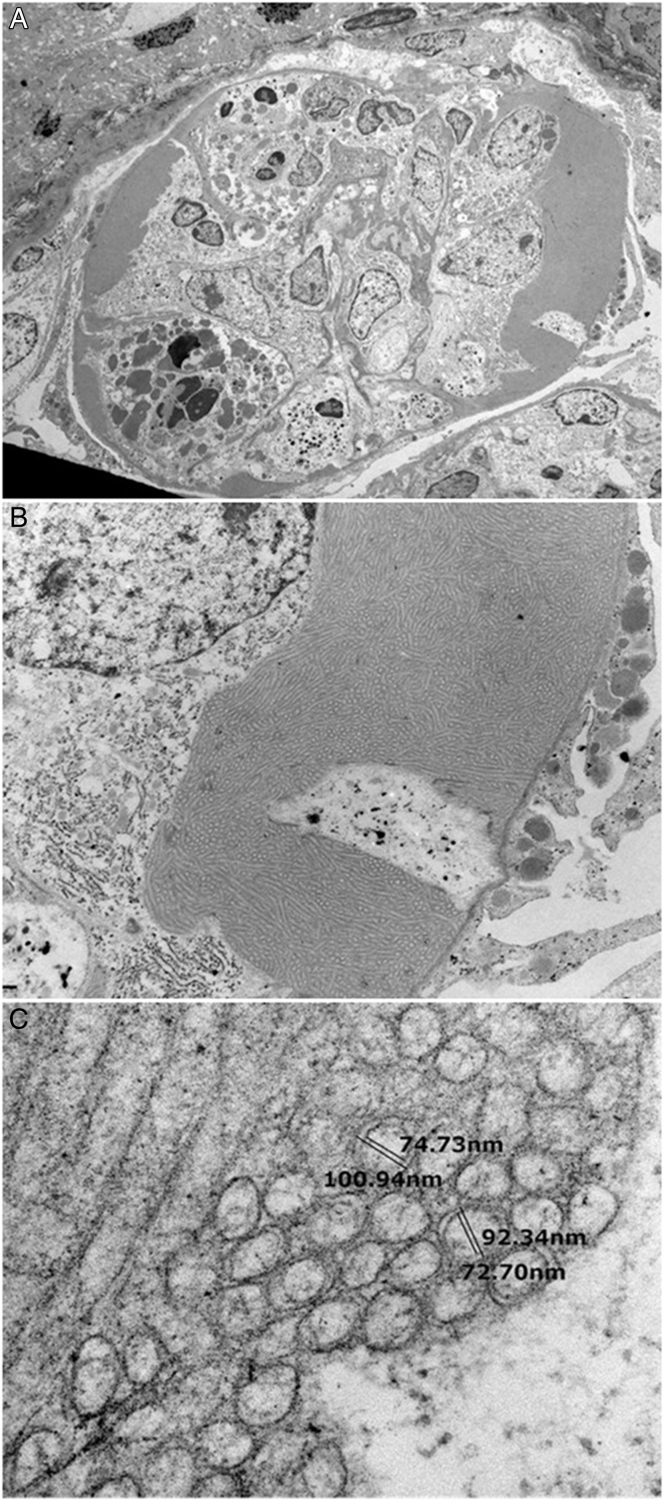
Electron microscopy of deparaffinized tissue. Top panel: Low magnification view of a glomerular capillary loop occluded by endothelial proliferation and leukocytic infiltration. Abundant electron-dense material is present within the subendothelial margins. Middle panel: Higher magnification view of the subendothelial material. Bottom panel: Very high magnification reveals the tubular nature of the material. In cross-section, the tubules display single and double walls. These microtubules were approximately 100 nm in outer diameter, with a hollow core consistent with cryofibrinogen.

Electron microscopy performed on deparaffinized tissue (Fig 2) showed that the mesangium, glomerular basement membranes, and podocytes were unremarkable. The glomerulus displayed 2 focal areas of leukocytic infiltrate with subendothelial accumulations of randomly oriented double-walled microtubules. These microtubules were approximately 100 nm in outer diameter with a hollow core, consistent with cryofibrinogen.

The patient was diagnosed with renal-limited cryofibrinogen glomerulonephritis without cryofibrinogenemia. Considering the significant degree of scarring with limited reversibility, and lack of extra-renal manifestations, no immunosuppressive therapy was administered. The small 2 g/L paraprotein was thought to represent monoclonal gammopathy of uncertain significance, and he has not yet had a bone marrow biopsy. He has been referred for chronic kidney disease education and dialysis planning.

## Discussion

To our knowledge, this describes the first case of cryofibrinogen-associated glomerulonephritis without a positive plasma cryofibrinogen.

Given the lack of plasma cryofibrinogen, the diagnosis was predominantly based on ultrastructural changes, namely double-walled microtubules of approximately 100-110 nm in diameter. This is supported by the histological and immunofluorescence findings of a membranoproliferative glomerulonephritis pattern with very rare glomerular intracapillary deposits in the absence of significant immunoglobulin and complement staining with segmental fibrinogen staining. The differential diagnosis from an ultrastructural point of view is immunotactoid and cryoglobulin; however, they are not typically double-walled or as large in diameter. Our case was not suggestive of a monoclonal gammopathy of renal significance due to the nonspecific immunofluorescent findings, in particular, the absence of kappa and lambda monoclonal restriction, including after protease digestion.^7^ Ultrastructurally, the electron microscopy findings did not show features supporting immunoglobulin deposits as well as no suggestions of monoclonal gammopathy of renal significance lesions without deposits, including thrombotic microangiopathy associated with monoclonal gammopathy. Monoclonal immunoglobulin deposits ultrastructurally show microtubules with diameters 17-52 nm,^8^ further consolidating our findings of cryofibrinogen-associated glomerulonephritis. Furthermore, our case is extremely unique due to the lack of plasma cryofibrinogen; thus, we were unable to perform any plasma mixing tests to evaluate the M protein as previous studies have done.^9^

The accuracy and sensitivity of cryofibrinogen detection is critically dependent on the method of collection and sample handling.^10^ As such, we repeated the blood cryoglobulin and cryofibrinogen sample 3 separate times each with a negative result, reducing the likelihood of a false negative. We ensured all samples were drawn into a prewarmed (37°C) anticoagulated (with ethylenediaminetetraacetic acid or citrate) tube and maintained at 37°C until centrifuged.

Cryofibrinogen-associated glomerulonephritis is an ultra-rare condition and thus, its prevalence is unknown. Moreover, there are no case reports of renal-limited cryofibrinogen disease and a scarcity of renal-limited cryoglobulinemic cases.^11^ Due to its complexity, investigating for a secondary cause of cryofibrinogen is critical. Up to 30%-50% are attributable to conditions such as autoimmune disease, infections, and solid or hematological malignancies.^12^ As outlined in our case report, a thorough screen for a secondary cause was undertaken and not found. In particular, the exclusion of infective endocarditis was prudent as our patient had a prosthetic heart value.

In our case, the ultrastructural appearance of large microtubular structures with linear fibrils in a matrix supported the diagnosis of cryofibrinogen. The arrangement and size of the microtubular structures assists with differentiation between cryofibrinogen, cryoglobulinemia, immunotactoid, and fibronectin glomerulopathy.^1^ They all share similar light microscopy features.

Due to a paucity of similar cases, we cannot hypothesize how plasma-negative versus positive cryofibrinogen-associated glomerulonephritis impacts prognosis. It is surprising that our patient had no other associated symptoms or signs suggestive of phenotypic cryofibrinogen vasculitis. We contemplated whether the long-standing use of warfarin for anticoagulation mitigated breakthrough cutaneous manifestations.

## Summary

This case presents the first instance of cryofibrinogen-associated glomerulonephritis without detectable plasma cryofibrinogen, with diagnosis based on ultrastructural findings. Differential diagnoses, including immunotactoid glomerulonephritis, were ruled out through morphological analysis. The case contributes to the limited literature on renal-limited cryofibrinogen and highlights the importance of comprehensive investigations, including electron microscopy of kidney biopsies, for accurate diagnosis.
